# Pathways to sexual decision making by Pentecostal church youths in Botswana

**DOI:** 10.1186/s12889-021-10645-8

**Published:** 2021-04-06

**Authors:** Elias Mpofu, Kayi Ntinda, Lisa Lopez Levers, Angelique van Rensberg, Fidelis Nkomazana

**Affiliations:** 1 Rehabilitation and Health Services Department, College of Health and Public Services, University of North Texas, 1151 Union Circle #, Denton, TX 311456 USA; 2 University of Sydney, Australia, Camperdown, Australia; 3 University of Johannesburg, South Africa, Johannesburg, South Africa; 4 University of Eswatini, Kwaluseni, Eswatini; 5 University of Botswana, Gaborone, Botswana; 6 Duquesne University, Pittsburgh, PA USA; 7 North West University, Vanderbijlpark, South Africa

**Keywords:** Abstinence; religiosity, Youth, Sexual decisions, Contraception, Personal control, Self-efficacy

## Abstract

**Background:**

The ways church youth make sexual decisions are incompletely understood and yet important for public health interventions. This study aimed to examine personal religiosity influences on the sexual decisions by church youth from the country of Botswana, taking into account their sense of personal agency.

**Method:**

Participants were 235 Botswana Pentecostal faith church youth (females = 67.2%, male = 32.8%; age range 12–23 years). They completed measures of personal religiosity, personal agency, sexual abstinence, and contraception use predisposition. We analysed the data applying Structural Equation Modelling to test five paths - personal religiosity to personal agency, personal agency to abstinence, personal religiosity to abstinence, personal agency to contraceptive use, and personal religiosity to contraceptive use.

**Results:**

Results suggest that personal religiosity influences the youth in their sexual abstinence and contraception decisions through personal agency. High personal agency, but not personal religiosity, was associated with pro-sexual abstinence, and contraception use was associated with religiosity. Personal agency augmented the likelihood of both abstinence and contraception use decisions among the older church youth and with church youth with higher levels of formal education.

**Conclusion:**

Church youth likely adopt discretionary sexual behaviours over the developmental period from early to older adolescents, which would make them more receptive to public sexual health messages. Personal agency appears to be an important resource for public health interventions aimed at influencing church youth’s sexual decisions.

## Background

Religion influences the sexual health behaviors of followers in many ways. For instance, some religions may teach sexual intercourse as a faith-regulated activity, and for those in a consummated relationship only [1–3]. They also would ascribe moral blameworthiness to members who infringe religious teachings upon rites to sexual unions [4, 5]. For instance, some church communities associate sexual intercourse outside of a consummated relationship with a “sin”, making sexual intercourse an emotive matter shrouded in taboo [6, 7]. For this reason, some church communities would apply considerable social sanctions on followers engaging in sexual activity that is contrary to faith teachings [8]. Regardless, some church followers self-report premarital sexual activity or engaging in sexual activity outside of a marital relationship [9], increasing their risk for sexually transmitted infections (STIs), including HIV [10–12]. With this evidence in mind, some church organizations increasingly collaborate with public health agencies in sexual health education in order to contain, if not eradicate STIs [13–15].

Sexually active church youth may carry a disproportionate risk for STIs from unprotected sexual encounters as emerging adults [16–22], perhaps due to lower exposure to public health education on contraception use [23]. For this reason, churches increasingly are mindful of the importance of developing partnerships with public health agencies, which subscribe not only to sexual abstinence, but also to the use of barrier contraception, within or external to a marital relationship [10, 18].

The youth’s personal sense of religiosity choices and decisions would likely influence their actual sexual behaviour as church followers, including abstinence and use of contraception [10, 24]. Primary abstinence refers to not having any history of penetrative sex. By contrast, secondary abstinence is refraining from penetrative sex with sexual debut or a history of prior penetrative sex [25]. Barrier contraception includes the use of condoms to prevent unwanted pregnancy or acquiring sexually transmitted infections (STIs). Youth with first-sex (or sexual debut) can elect secondary abstinence with or without a romantic partner, reducing their risk for unwanted pregnancy and STIs [24]. They may perceive that they have personal agency or the ability to control their social outcomes and their health outcomes [10].

While church followers, people may dissect their behaviors between those they perceive to be of their own volition and those they perceive to be by their religion teachings [26, 27]. In other words, the youth’s personal sense of religiosity choices and decisions would likely influence their actual sexual behaviour as church followers [5, 28, 29]. However, it is unclear from the literature how church youth make personal choices about their sexual activities in the context of adhering to the tenets of their religion. However, it is unclear from the literature how church youth make personal choices about their sexual activities in the context of adhering to the tenets of their religion. We aimed to address this knowledge gap by examining how personal religiosity and personal agency influence sexual abstinence and contraceptive use decisions by Botswana Pentecostal church youth, a country in which 25% of the church youth reported having engaged in premarital sex [10, 24].

## Literature review/theoretical framing

Church historical sexual teachings create a culture that provides continuity for present practices by followers [5]. This view is consistent with Cultural historical activity theory (CHAT: [30, 31]), Church endorsed knowledge influences the member actions through collective learning, which permeates followers’ shared organization consciousness. Followers them make choices framed on historical learning, which is reinforced by ongoing teachings so members become selectively receptive to institution supported knowledge values and ways of knowing, subordinating alternative, valid understandings [31]. In the context of sexual decisions, church historical teachings be that unmarried members adhere to church teachings of sexual abstinence, [10], which would obviate use of contraception. Adherence to sexual abstinence teachings explains why church youths would be less likely to engage in causual sexual intercourse or to have teenage pregnancies, suggesting a robust religiosity context effect [5, 27]. Nonetheless, church youth believing that they are good followers self-report with premarital sex, while also perceiving themselves to be church followers in good standing [10, 32]. This suggests nuanced decisional influences by church youth, who are in a developmental stage, and working on their evolving identifies as followers of a faith. Prospectively, how church youth understand their personal religiosity and personal choices to influence their sexual decisions is the goal of this study.

### Personal religiosity and personal agency influences on sexual decisions

Personal religiosity refers to one’s self-beliefs as a follower of a particular religion [2]. It is associated with a sense of identity with other religious practices, including actionable beliefs about appropriate health behaviors, including sexual decisions [26–28]. An intrinsic religion adherence, rooted in conviction, would be binding to church moral teachings, endorsing sexual abstinence until married [28], and few would use contraception [23, 29]. By contrast, an extrinsic religion adherence, one that prioritizes social membership, perhaps with less personal conviction, would be more open to sexual experimentation and use of condoms for contraception [10, 32]. If this were the case, a sense of personal agency would explain how their individual religiosity orientation translates into the choices they make regarding sexual abstinence and use of contraception.

Following socio-behavioral theory, personal agency is framed in two parts: perceived control and self-efficacy [27, 33]. Perceived control refers to a belief in self-determining one’s own actions, while self-efficacy refers to the belief in one’s ability to achieve specific outcomes. High personal agency is associated with making choices to forge a difference in one’s own life. It is generative and proactive in nature, rather than reactive. For instance, people with a higher sense of personal agency are more likely to adopt elective health promotion activities than those with a lower sense of personal agency [27, 32]. Consequently, people with low personal agency carry health-compromising risks because of not engaging in health promotion actions [34, 35]. It is unclear how personal religiosity and personal agency would influence sexual decision making under the homogenizing influences of church following.

In theory, church youth with high personal agency would choose to be guided in their sexual decisions by both faith-led and public health teachings [5], while those with low personal agency would defer to obligatory faith teachings alone [5, 36]. Youth with low personal agency likely would engage in impulsive decision making, based upon self-perceptions of having a lack of personal control over their social outcomes [28, 37, 38].

### Goals of the study

Given the above set of assumptions drawn from the literature, we sought to address the following research question: How are personal religiosity (i.e., intrinsic-extrinsic orientation) and personal agency associated with explaining self-reported sexual abstinence and contraception use among Botswana Pentecostal church community youths. In exploring these relationships, we considered the moderating role of personal demographics (age, sex, education).

## Method

### Participants and setting

We derived the methodological framework for this cross-sectional study from a larger study on sexual decision making and HIV/AIDS prevention among Pentecostal church youth in Botswana [10, 39]. The larger study utilized concept mapping approaches [39] to construct the components and content for a Botswana Pentecostal church-supported HIV/AIDS prevention curriculum for use with the youth congregates. The data for this study were a part of the larger study aimed at validating the outcome measures to be used in a subsequent preclinical intervention pilot study.

A strength of the larger study is that we developed a strong partnership with the Pentecostal church organization, successfully implementing a preliminary study with them to inform our larger study to follow. A limitation of the larger study is that we chose to work with only one of the Pentecostal churches in the country; there are doctrinaire differences among Pentecostal churches, and we were not able to observe them all. Botswana is a sparsely populated Southern African country of about 2.25 million people, and yet with one of the highest HIV infection rates in the world, at 21.9% [12]. Young people (15–24 years) comprise 18.91% of the population and carry 24.9% of HIV infections, making them the most at-risk population [39]. The high HIV prevalence among Botswana youth exists, despite the fact that about 80% of Botswana youth are Pentecostal church followers [40]. The Botswana Pentecostal church primarily advocates sexual abstinence and sexual fidelity, although their endorsement of condom-promotion as a public health intervention is on the increase [18].

Participants in the study were 235 youth (female = 67.2%, male = 32.8%; age range 12–23 years) from a major Pentecostal church in Botswana comprising 26 congregations. Table 1 presents the demographic information of the participants in this study.

**Table 1:** Participant demographics

Demographic	N	%
Sex		
Female	158	67.2
Male	77	32.8
Age		
12–15 years	36	15.3
16–19 years	86	36.6
20–23 years	111	47.3
Singles	149	63.4
With first sex	62	26.3
HIV/AIDS identity		
Orphans	58	24.6
Living with HIV	51	21.7
Congregation Location		
Urban	172	73.1
Rural	63	26.8
Education level		
Primary-Junior	59	25.1
High School	91	38.7
Tertiary	85	36.1

Note. Singles = not in a romantic relationship

We selected the participants in two stages. First, we used proportional random sampling to select eight of the 26 congregations (30%), from across the country, for study: three large congregations (500 or more followers), two medium-size congregations (250–499 followers), and three smaller congregations (less than 250 followers). Proportional random sampling of at least 30% of eligible units is adequate for reliable parameter estimations [41]. Of these congregations, five were in urban areas, and three were in rural areas. Second, we then employed simple random sampling (using a table of random figures) to select the youth for study from the congregations already identified for the investigation. The youth had been involved with the Pentecostal church for an average period of 5 years (SD = 4.93 years).

Our Pentecostal church partner, in this inquiry, was typical of others of the same faith in Botswana in terms of youth enrolment and geographical dispersion. The church follows a prosperity gospel doctrine of a benevolent, health-gifting God. All of their congregations follow the same church teachings, delivered by pastors and elders who hold membership in a national church council, thereby ensuring doctrinal uniformity. For this reason, we assumed that there would be no differences in church doctrine teachings across the church’s congregations.

This particular Pentecostal community has a strong regional presence in sub-Saharan Africa with significant followings in West Africa (i.e., Ghana, Nigeria) and Southern Africa (i.e., Namibia, South Africa, Eswatini (formerly Swaziland), Zambia, and Zimbabwe). Findings from this study would likely be indicative of church youth decisional influences among Pentecostal faith congregations in the sub-region.

### Measures

The church youth completed the Botswana Youth Health Survey (BYHS) [42]. The BYHS includes a biographical measure, encompassing age-in bands (12–15 years, 16–19 years, 20–23 years), sex, length of time affiliated with congregation and Pentecostal church, sexual debut, relationship status (single versus in a relationship with other), place of residence, level of educational attainment, and HIV status identity (orphaned by HIV, living with HIV). The BHYS also includes Likert-style questions on personal religiosity, sexual abstinence, and contraceptive use. We developed and piloted the survey on a sample of church youth as part of the larger study, which focussed on aspects of religiosity that influence HIV prevention among Pentecostal Botswana teenagers [32]. In doing so, we conducted a *Setswana* translation of the survey by forward translation methods from English to Se*tswana* for use with those participants who preferred to take the surveys in *Setswana* (the local language). English is the medium of instruction in Botswana schools from pre-school to higher education, while *Setswana* is a written language also taught in Botswana schools.

We developed the BYHS items beginning with open-ended questions, the responses to which resulted in rating scale type of measures. In addition, evidence from the literature also guided our conceptualization and generation of items. Finally, we selected best-fitting items for each of the measures from the results, following exploratory factor analysis and confirmatory factor analysis. We desribe these measures below.

#### Sense of personal agency

We adapted eight items related to personal agency from the study by Donohew et al., [43]. The items are scored on a 5-point Likert scale, from *Strongly disagree (1)* to *Strongly agree (5)*. Examples of the pro-agentic items from this measure include the following: “I think carefully about actions I do, rather than do the first thing that comes into my mind”; “I do things that would help me in the long term, rather than whatever I think will be most fun”; and, “I take my time to do things, rather than act on things as soon as I think about them.” Higher scores indicated a higher sense of personal agency. In the present study, we observed an internal consistency score of 0.73 for scores from the *Sense of personal agency* measure.

#### Personal religiosity

Our personal religiosity measure was comprised of two subscales: Extrinsic personal religiosity (EPR = 4 items) and intrinsic personal religiosity (IPR =5 items). Response options ranged on a 3-point scale (0 = *Never*, 1 = *Sometimes*, and 2 = Q*uite often*). We measured Extrinsic personal religiosity by self-reported frequency of participating in the following activities: church services, church choir, Bible study, fundraising activities, charity activities, and visiting the sick/bereaved. We measured Intrinsic personal religiosity by the frequency of participation in meditation, prayer (non-specific), prayer for guidance that others ‘do the right thing,’ prayer for God’s forgiveness for personal/others’ misdeeds, prayer for receiving God’s blessing, and prayer for God’s blessing for others. The internal consistency of scores from the *Personal religiosity measure* for this study were moderate to high (EPR: 0.73 and IPR: 0.91).

#### Abstinence

We measured for both Primary Abstinence (PA Scale; 4-items) and Secondary Abstinence (SA Scale; 4 items). Items on both scales are scored on a 5-point Likert scale ranging from *Strongly disagree (1)* to *Strongly agree (5)*. The PA subscale is a measure of sexual attitudes as would be endorsed among youths with no sexual debut. Examples of items from the PA scale are as follows: “I do not want to get a disease,” “I am waiting to have sex until I get older,” and “I would be embarrassed.” We used the first sex question as a filter, so that church youth reporting their first sex would then skip the PA scale questions to answer the SA scale questions.

The SA subscale is a measure of sexual predispositions or behavioral intention to indulge in sexual intercourse. Items include the following statements: “My friends think it is wrong to have sex at our age,” “I don’t have a boyfriend or girlfriend to have sex with,” “My boyfriend or girlfriend does not want to,” and “I couldn’t get birth control or protection.” All youth responded to the SA scale. The internal consistency of scores from the *Abstinence measure* for this study were high (PA: *alpha* = 0.82 and SA: *alpha* = 0.77).

#### Contraceptive use

The Contraceptive Use (CU) subscale is comprised of seven items on a 5-point Likert scale: *Strongly agree* (1) to *Strongly disagree* (5). Sample items from this measure include the following: “Said no to sex without a condom,” “Easy for me to ask a partner to use a condom,” and “Talk to partner about sexual intercourse.” We observed an internal consistency index of 0.75 for scores from the CU measure in the present study.

### Procedure and ethical considerations

The Human Research Ethics Review Boards of the University of Sydney and the University of Botswana’s Office of Research and Development approved the study (Protocol # FHS 11501). The parents or guardians of the church youths who were minors (< 16 years of age) consented in writing for their children to take part in the study. In addition, the church youth who were minors individually assented in writing for study. The youth of legal age to consent (16 years or older) individually consented to the study in writing. We described the study goals to the participants in writing and as part of the study consent procedure. We also informed the participants in writing of their right to discontinue participation of the survey study at any time and with no penalty. We also assured the participants of both the confidentiality and anonymity of their data. Participants individually completed the survey at their church centre and during times mutually agreed upon by the research team and the church organization. As a token of appreciation, participants could keep the pens they used to complete the survey. Less than 2% of the randomly selected participants declined, citing competing activity at the time of the surveys.

### Data analysis

For the analysis, we utilized Latent variable modelling in Mplus 7.4 and a maximum likelihood (ML) estimator [44]. We specified our measurement model to answer the question: *How are personal religiosity (*i.e.*, intrinsic-extrinsic orientation) and personal agency associated with explaining self-reported sexual abstinence and contraception use among church community youths*? In the structural model, we tested the following five paths: personal religiosity to personal agency, personal agency to abstinence, personal religiosity to abstinence, personal agency to contraceptive use, and personal religiosity to contraceptive use.

We estimated missing values with full information maximum likelihood (FIML) function. Statistics were evaluated to establish model fit and included chi-square (χ^2^), Tucker-Lewis Index (TLI), comparative fit index (CFI), root mean square error of approximation (RMSEA), RMSEA 90% confidence intervals (CIs), and standardized root mean square residual (SRMR). TLI/CFI scores greater than .90 and RMSEA, RMSEA 90% CI smaller and SRMR scores smaller than .08 (*p* > .05) indicate close fit to the model [45]. Since tau-equivalence was violated (i.e., that every item contributed equally to the latent variable), Cronbach’s alphas were not employed, but rather, we calculated point-estimate reliabilities (ρ) following the procedures by Raykov [46].

We further conducted mediation analysis, using factor scores calculated for each latent variable, with Process macro for SPSS Release 2.16 in IBM SPSS Statistics 24 [47, 48]. To achieve this, we utilized factor scores, obviating the need to centre the mediator and/or independent variables [49]. We also corrected for possible confidence interval bias at the upper level (ULCI) and lower level (LLCI) of the factor score ranges. Moreover, we tested for whether age band, sex, or educational level had a moderating effect on the significant regression paths identified in the structural model. Finally, we analyzed the data from interaction plots to determine any significant moderation effects.

## Results

Table 2 presents the correlational analysis and descriptive statistics of the study variables. As apparent from Table 2, personal religiosity and sense of personal agency were significantly associated with secondary abstinence beliefs about making safer sex decisions (*p.* <. 01). While intrinsic and extrinsic religiosity were significantly and positively related, their inter-correlations were low (r < .36), suggesting that they measured uniquely different facets of the personal religiosity variable. The correlations between personal agency and personal religiosity scores were low (less than r = .34), supporting their use as unique predictors of the sexual decisions made by the church youth. The sex of church youth was not significantly associated with any of their sexual decisions pertaining to abstinence, contraction, personal religiosity, or personal agency, thereby indicating a need to analyze for the total sample without splitting by sex. Age at first sex was 17.72 years (SD = 3.57), with 66.1% reporting to have consented. Among those making their sexual debut, the decision to use contraception was associated with an extrinsic religiosity orientation (r = .39; *p.* <.01).

**Table 2:** Correlations and descriptive statistics of the study variables

Variable	1	2	3	4	5	6	7	8	9	10	Mean	SD
1.Primary abstinence	–										2.60	1.02
2.Scondary abstinence	−.01	–									4.38	.79
3.Contraception	.21	.24^*^	–								3.14	1.24
4.Personal agency	.08	.41^**^	.14	–							4.15	.70
5.Extrinsic religiosity	.19	.26^**^	−.07	.26^**^	–						1.23	.43
6. Intrinsic religiosity	.16	.24^**^	−.04	.28^**^	.35^**^	–					1.66	.33
7.Sex	.02	−.07	−.03	−.10	.00	−.01	–				–	–
8.Age	.45^**^	.05	−.14	.09	.30^**^	.26^**^	.03	–			–	–
9. Singles	.08	.06	−.16	.20	.09	.04	.02	−.03	–		–	–
10. First sex	–	.15^*^	.46^**^	−.04	.17^*^	.13	.08	.39^**^	.03	–	17.72 yrs	3.52
N	123	216	114	194	163	181	225	223	141	225	–	–

Note. ^*^p <. 05, ^**^p < .01; Single =0; In relationship =1; First sex = only those with sexual debut

As would be expected, we observed a significant, positive correlation between youth with first sex and their use of secondary abstinence as well as contraception use (*p.* <. 05). Moreover, we observed a significant, positive association between youth with first sex and their extrinsic religiosity scores.

### Modeling personal agency and personal religiosity in sexual abstinence and contraception use

**Fig. 1:**
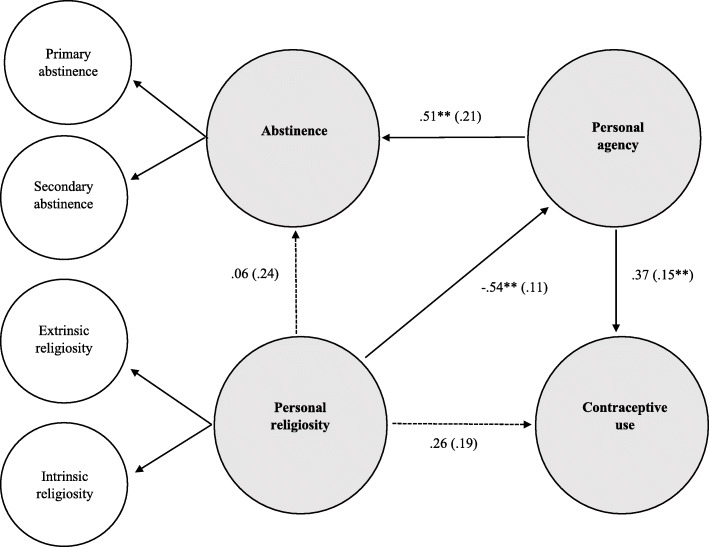
Structural model of contraption use with sexual abstinence, personal agency and personal religiosity (The dashed lines represent the non-significant paths/Solid line the significant associations)

Following the Structural Equation Modeling [SEM) (see Fig. 1), we observed two first-order latent variables (i.e., contraceptive use [α = .75] and personal agency [α = .74]), as well as 2 second-order latent variables (i.e., abstinence [scale 1: α = .82; scale 2: α = .77]) and personal religiosity (scale 1: α = .73; scale 2: α = .91). All latent variables indicated acceptable point-estimate reliability; hence, we allowed for their correlation. The fit indices showed acceptable fit (χ^2^ = 487.51, *p* < .05, CFI = .93, TLI = .93, RMSEA = .03, 90% CI [.02, .04], *p* > .05, SRMR = 0.08), lending confidence to the model being a reliable representation of the data and not requiring re-specification.

The results from the SEM further indicated that personal agency and abstinence (β = .48, *p* < 0.05) and personal agency and personal contraceptive use (β = .23, *p* < 0.05) were positively related. Moreover, higher total personal religiosity scores were significantly negatively correlated with personal agency (β = −.54, *p* < 0.05).

### Pathways to endorsement of contraception use by church youth

We based our structural model on the measurement model (as in Fig. 1). To clarify the role of personal religiosity as well as personal agency in participants’ self-reported abstinence and contraceptive use, we placed the correlations between the latent variables by their paths/regression coefficients. To achieve this goal, we tested a structural model with the following five paths: personal religiosity to personal agency, personal agency to abstinence, personal religiosity to abstinence, personal agency to contraceptive use, and personal religiosity to contraceptive use (see Fig. 1 and Table 3). Fit statistics indicated acceptable fit (χ^2^ = 487.51, *p* < .05, CFI = .93, TLI = .93, RMSEA = .03, 90% CI [.02, .04], *p* > .05, SRMR = 0.08), suggesting that the model is reasonably consistent with the data. However, the regression coefficients (β) indicated that only these three significant paths were present: (a) when young people had higher total personal religiosity scores, their personal agency scores were lower (β = −.54, *p* < .05); (b) youth with higher personal agency scores had higher self-reported abstinence scores (β = .51, *p* < .05); and, (c) young people who scored higher on personal agency also had higher self-reported contraceptive use (β = .37, *p* < .05).

**Table 3:** Moderation analyses

	Coeff	SE	t	***p***	LLCI	ULCI
**Personal agency ➔Abstinence**						
Age	−0.05	0.04	−1.54	0.13	−0.13	0.02
Personal agency X Age	0.07	0.04	1.49	0.14	−0.02	0.15
Gender	0.02	0.04	0.53	0.60	−0.06	0.10
Personal agency X Gender	0.03	0.06	0.45	0.65	−0.10	0.16
Education level	−0.08	0.03	−2.92	0.00^*^	−0.13	−0.03
Personal agency X Education level	0.11	0.07	3.96	0.00^*^	0.05	0.16
**Personal agency ➔Contraceptive use**						
Age	0.07	0.03	2.16	0.03^*^	0.01	0.14
Personal agency X Age	0.01	0.04	0.29	0.77	−0.07	0.10
Gender	0.00	0.04	−0.11	0.91	−0.08	0.07
Personal agency X Gender	0.06	0.06	1.00	0.32	−0.06	0.18
Education level	−0.02	0.03	−0.89	0.37	−0.08	0.03
Personal agency X Education level	0.00	0.03	−0.04	0.97	−0.06	0.05
**Personal religiocity ➔Personal agency**						
Age	0.08	0.10	−1.75	0.08	−0.39	0.02
Personal religiocity X Age	−0.20	0.19	−1.04	0.30	−0.58	0.18
Gender	0.02	0.05	0.33	0.74	−0.08	0.11
Personal religiocity X Gender	0.10	0.28	0.34	0.73	−0.46	0.66
Education level	0.10	0.03	0.42	0.68	−0.05	0.08
Personal religiocity X Education level	0.11	0.15	0.73	0.47	−0.18	0.39

^*^ Note. *LLCI* Lower level confidence internal, *ULCI* Upper level confidence interval; Level of confidence for all confidence intervals were 0.05

### Mediation of sexual abstinence and contraception use by personal agency

Analyses derived from the SEM indicated ted the following three significant paths: personal agency to abstinence, personal agency to contraceptive use, and personal religiosity to personal agency (see Table 3). We computed the mediation analyses separately, specifying non-essential variables as covariates (i.e., items not specified as independent, dependent, or moderating variables). Results indicated that higher education level was a significant predictor of lower abstinence scores, while being an older youth was a significant predicator of higher contraceptive use (see Table 3). However, higher education level had a significant mediation effect on the relationship between personal agency and abstinence.

The results of the interaction plots indicated that personal agency and sexual abstinence were significantly associated among church youth with higher levels of formal education, compared to those with lower levels of education. This means that the presence of personal agency and education level are significant in the prediction of higher sexual abstinence scores.

## Discussion

Results from this study suggest that personal religiosity has little direct effect on the sexual abstinence and contraception-use decisions made by church youth. This was a rather surprising finding, in view of the emerging evidence regarding personal religiosity influences on sexual abstinence [24, 25, 28]. We expected, on the basis of Cultural Historical Activity Theory (CHAT: [30, 31]), that high personal religiosity would have had a direct influence on sexual abstinence, especially as church teachings regard sexual abstinence to be strictly obligatory, mandating that sexual choices be consistent with divine guidance [36]. It seems that this effect would bolster self-control in the face of hedonistic sexual urges [27]. Rather, personal religiosity influenced the church youth’s sexual abstinence and contraception use through their sense of personal agency. This suggest that homogenizing influences of church teachings based on historical teachings may not directly influence sexual decisions of church youth they perceive to be of their choice.

Consistent with socio-cognitive theory [27, 33], perceived self-control as an aspect of personal agency, would mediate the relationship between personal religiosity and sexual abstinence and contraception use decisions [5, 27]. For instance, the church youth may choose to act differently from religious teaching, aligning their sexual behaviors to their private personal or social network values [10, 28]. “Individuals may construct a domain of personal jurisdiction, in which clear limits to authority intrusion into the personal sphere are drawn (reflecting autonomy), while at the same time evincing a strong commitment to moral and social conventional norms (indicating relatedness)” ([50]; p. 470). In this regard, church youth with high personal agency may adopt elective health norms that draw from both religion and from others in their social network [5, 29]. In doing so, young people may be motivated by the the personal drive to maintain and enhance their social status within the peer group [10, 32], even if their health choice actions may differ from those promulgated by faith teachings [5, 51]. There is emerging evidence to suggest that some social segments existing within a faith community may elect to prioritize secular health actions rather than actions based on religious belief [29, 52]. “Autonomy, conceived as agency or volition, does not necessarily imply separation from others” [50], p. 460. This being the case, while personal religiosity might frame the context in which church youths make sexual decisions, it would not directly explain the church youth’s sexual decisions (see also [9, 23, 34].

Church youth with higher levels of education had lower levels of sexual abstinence scores, suggesting a moderation effect of formal education on the youth regarding their sexual behaviors. Higher formal education also is associated with personal agency across a range of life outcomes, including sexual behaviors [20, 53, 54]. The *Botswana Skills for Life* educational program [55], used in junior and secondary schools nationwide, provides education on sexual decision making choices of being abstinent, on remaining faithful to a partner if sexually active, and on using contraception during sexual encounters.

Not surprisingly, church youth with higher levels of education would likely adopt discretionary, interpretive sexual health norms [5, 10, 39], motivated by their sense of personal agency, thus reconciling their religious teachings and the public health education that they received from school, including use of contraception. On the one hand, the church youth with higher levels of education would sexually indulge, with the use of contraception, following secular education practices. On the other hand, church youth with higher levels of education may choose to abstain from sex, perceiving a higher sense of personal agency from their secular education [5]. They also choose to indulge in sexual activity if they believe it to be one aspect of a thriving spiritual life with God, and in a fulfilling sexual relationship [2, 4]. Arguably, church youth with lower education and strict adherence to faith teachings may abstain from sex. However, they would be more susceptible to unsafe sexual practices due to a lack of knowledge about their contraception options [9, 23, 28].

Older age church youth endorsed the use of contraception more than the younger church youth. This result can be explained by the fact that the older church youth in the faith community under study were likely to be sexually active [10]. The findings also can be explained by the fact that older youth might have a higher sense of personal agency, due to maturation and development [41]. Helwig [50] indicates that personal agency places “limits on the legitimate actions of authorities and other social agents” (p. 459). Older age church youth also would have higher exposure to secular education, which prioritizes private, individual choices in the context of relationship practices [10, 56].

### Implications for public health promotion with church youth

By implication, abstinence education alone is not enough (see also [9, 52]). Sexual health promotion activities with church youth need to take into account how the centrality of personal agency influences the sexual choices of church youths. Personal religiosity, while important to making sexual decisions, might not necessarily suffice in obviating sexual health risks for church teenagers with a lower sense of personal control in their sexual decisions [9, 34, 51]. For instance, Basinga et al., [57] reported no differences by type of school on contraception use among teenagers attending a secular school and a Baptist church school in Rwanda, a country with similar socio-demographics as Botswana. This was particularly true among sexually active church youths who framed their sexual health differently from the abstinence-only church teachings.

Historically, religious organizations are less likely to promote contraception use by their youth members, although this might be changing for some church organizations [18, 58]. While sexual decisions that favor abstinence and contraception use by church youth ultimately would rest upon their personal choices, church youth could elect to maintain commitment to their faith community beyond that premised on adherence to abstinence-only teachings [9, 28]. As they mature and increasingly realize influences of personal agency on human behavior, church youth who may have broken with sexuality teachings might perceive their actions as being justified by the unconditional love of God, inclusive of those who have transgressed [29, 59]. In other words, church youth may recalibrate to less adherence to church teachings following their first sexual experience [32, 60, 61], implying more personal agency in their sexual decisions. Hence, church youth would likely benefit from public health interventions that are framed within a concordant sense of personal agency along with their developmental period (early to late adolescence), rather than religious following only.

### Limitations of the study and suggestions for further research

Limitations of the study include the fact that this was a cross-sectional study, conducted with a single Pentecostal church in Botswana, which constrains the generalizability of findings. Future studies should employ a longitudinal design to determine the stability and change in personal religiosity influences on abstinence and contraception use over time. A longitudinal study also would allow for study of the frequency of secondary abstinence and contraception use among the sexually active church youth, in order to inform the design of targeted interventions with them. Our sample size yielded small cell numbers for the age by sexual initiation and education levels to allow for meaningful analysis by those interactions. Moreover, the study design over-relied on self-report data concerning personal religiosity and personal agency by church youths, rather than on data gathered from their actual church activity involvement and sexual decision histories. It is possible that some of the church youth under-reported their sexual activity decisions out of social desirability, even though we provided data confidentiality and anonymity assurances. Moreover, church youth appear to be on a continuum of intrinsic to extrinsic personal religiosity, necessitating further studies on the permutations within and between religiosity orientations and a concomitant sense of personal agency. While high religiosity has been associated with low personal agency [36], in theory, high personal agency is possible with either religiosity orientation. Use of receiver operating characteristic curve analysis [62] might help to indicate the tipping point in the youth religiosity and personal agency orientations for sexual abstinence and contraception use choices. Moreover, we utilized a measure of personal control in impulsive actions for personal agency rather than one of self-efficacy. This had the effect to under-operationalize the personal agency measure. Future studies could employ multi-dimensional measures of personal agency to examine, in detail, its specific effects on sexual decision making among church teenagers. There is need for prospective studies on personal agency and religiosity orientations (intrinsic vs extrinsic) effects on sexual abstinence statuses (primary and secondary) and contraception use to inform a pre-clinical intervention design study with a church youth population.

## Conclusion

The findings of this study suggest that personal agency has an influence on Botswana Pentecostal church youth’s sexual decision making, over and beyond their personal religiosity. This influence may be stronger among the older church youths, who possess a higher level of formal education. Moreover, the older church youth and those with higher levels of formal education may choose secondary abstinence and contraception use to best meet their needs. These findings suggest that religious orientation is subordinate when compared to the interaction of church youth’s sexual decision making and their sense of personal agency. The church youth’s sense of personal agency appears to be an important resource for public health interventions aimed at influencing their sexual decisions. Incorporating the construct of personal agency into existing public health education for youth in Botswana could play an important role in mitigating the further spread of HIV/AIDS in a county that continues to experience one of the highest incidence rates in the world.

## Data Availability

The data for this study are available from the lead author with reasonable request and following standard data management processes, and with assurances to use for study purposes only.

## Abbreviations

AIDS: Acquired Immune Deficiency Syndrome
BYHS: Botswana Youth Health Survey
CFI: Comparative fit index (CFI)
CI: Confidence Interval
CHAT: Cultural historical activity theory
CU: Contraceptive Use
EPR: Extrinsic personal religiosity
FIML: Full information maximum likelihood
HIV: Human Immunodeficiency Virus
PA: Primary Abstinence
IPR: Intrinsic personal religiosity
LLCI: Lower level confidence interval
ML: Maximum likelihood
RMSEA: Root mean square error of approximation
SA: Secondary Abstinence
SD: Standard deviation
SPSS: Statistical Package for the Social Sciences
STIs: Sexually Transmitted Infections
SEM: Structural Equation Modeling
TLI: Tucker-Lewis Index
ULCI: Upper level confidence interval
